# Supplementary material for: Integrated Multi-Omics Profiling Reveals That Highly Pyroptotic MDMs Contribute to Psoriasis Progression Through CXCL16
Source: Biomedicines. 2025 Jul 18;13(7):1763. doi: 10.3390/biomedicines13071763 (PMC12292276; doi:10.3390/biomedicines13071763)
Supplement: Supplementary file 1 [file biomedicines-13-01763-s001.zip › biomedicines-3686690-supplementary.pdf]

# Supplementary Information for

## Integrated Multi-Omics Profiling Reveals That Highly Pyroptotic MDMs Contribute to Psoriasis Progression Through CXCL16

**This file includes:**

**Figure S1-S5**

**Table S1-S7**

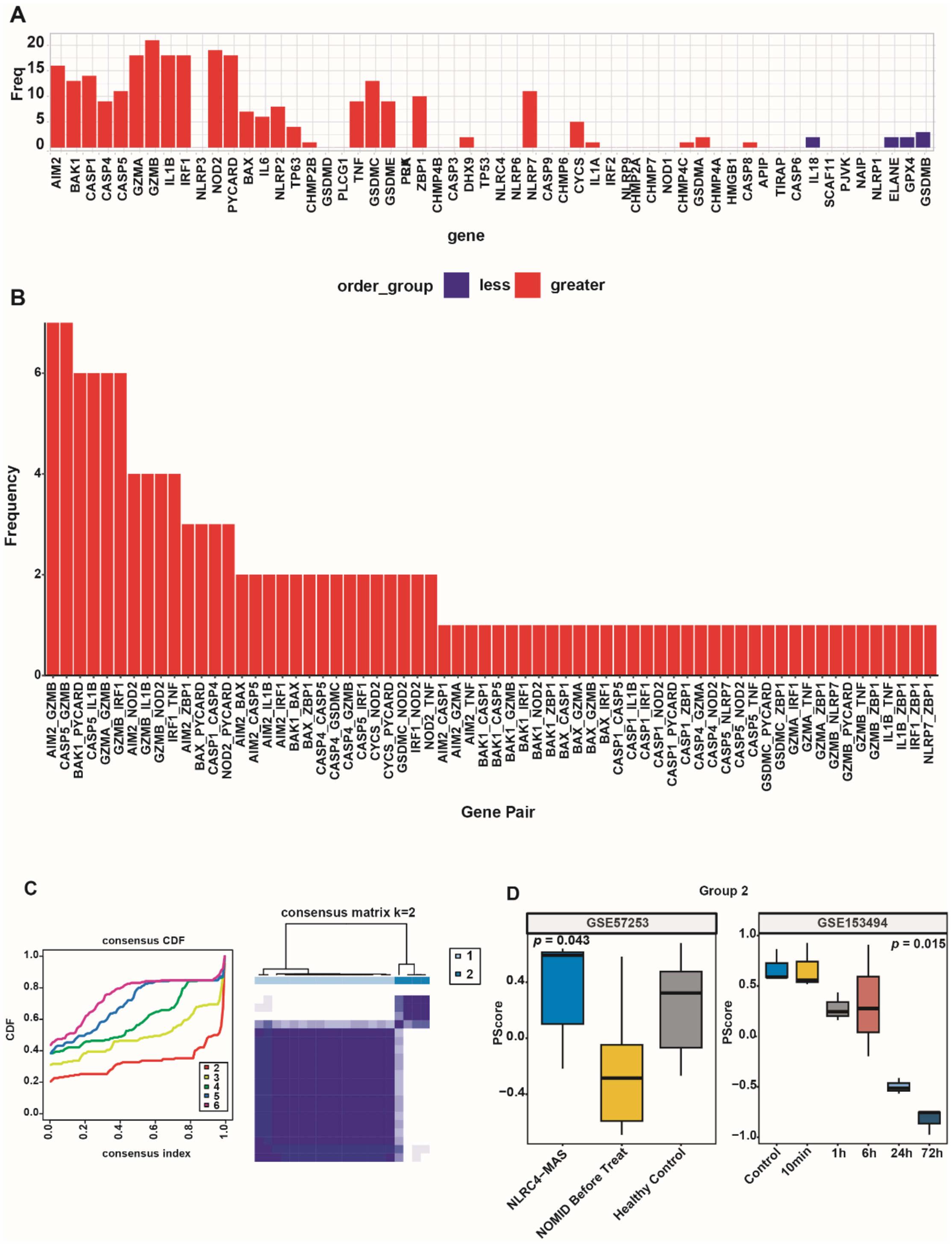


**Figure S1 Association of pyroptosis with psoriasis and development/validation of the pyroptosis score.** (**A**) Bar plot showing the frequency of differentially expressed pyroptosis-related genes in each dataset. (**B**) Frequency table of significant correlations (r > 0.8, $p \leq 0.05$) between pairs of the 20 pyroptosis-related genes. (**C**) Consensus clustering heatmap and corresponding CDF plot (Euclidean distance, Pearson correlation). (**D**) Box plot validating the GSVA scores of Group 2 pyroptosis-related genes in independent datasets (GSE57253 and GSE153494).

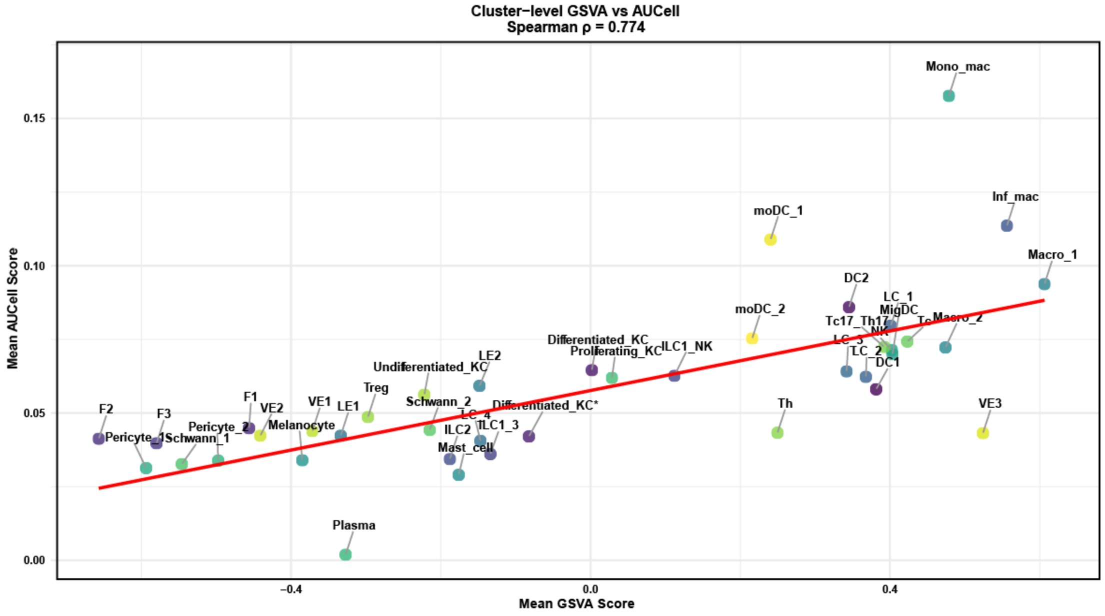


**Figure S2 Scatter plot showing Spearman correlation between mean GSVA enrichment scores and mean AUCell values across annotated cell subtypes ( ρ = 0.774, *p* <0.001).**

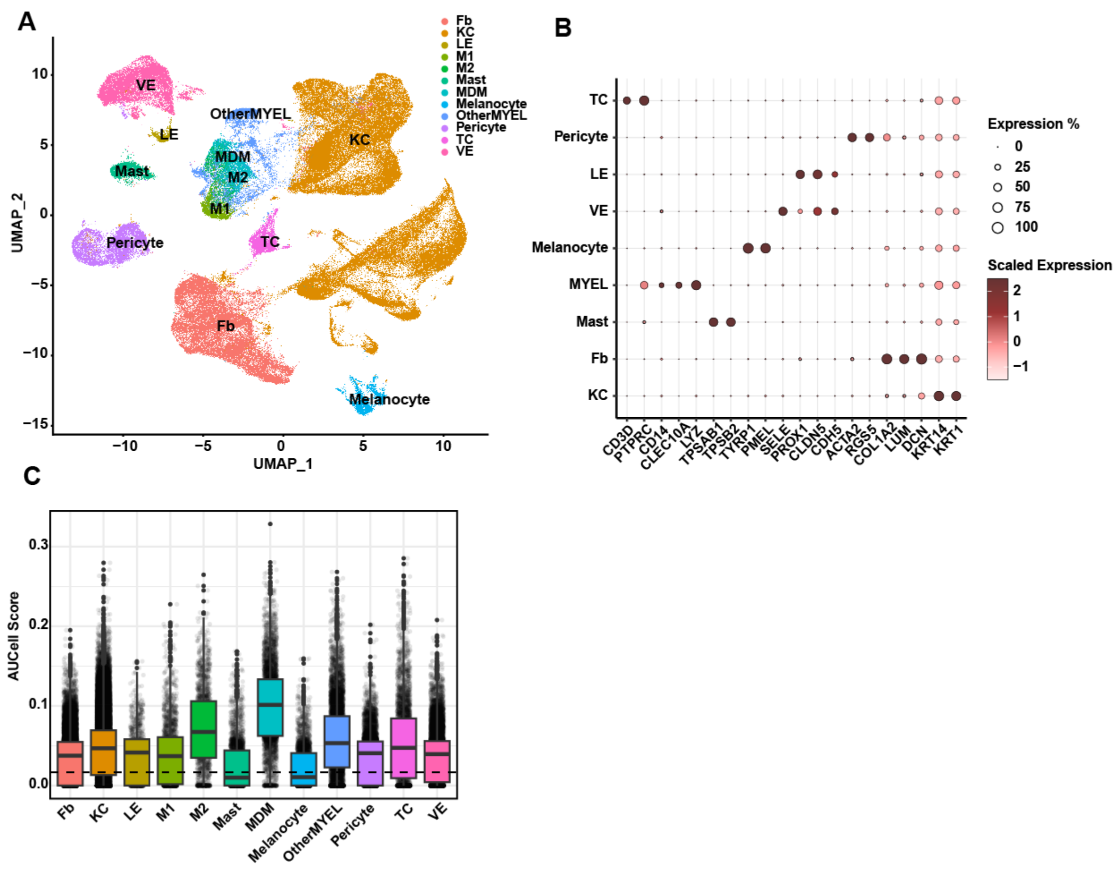


**Figure S3 Integrated single-cell RNA sequencing analysis of lesional skin reveals distinct cellular subpopulations and pyroptosis activity (GSE228421). (A)** UMAP dimensionality reduction of scRNA-seq data identifies cellular subpopulations in psoriatic skin (GSE228421). (**B**) Expression of canonical marker genes across identified cellular subpopulations. (**C**) Pyroptosis scores across cellular subpopulations evaluated by AUCell. VE, Vascular endothelial cells; TC, T cells; MYEL, Myeloid cells; MDM, Monocyte-derived macrophage; M, maceophages; LE, lymphatic endothelial cells; KC, Keratinocytes; Fb, Fibroblast.

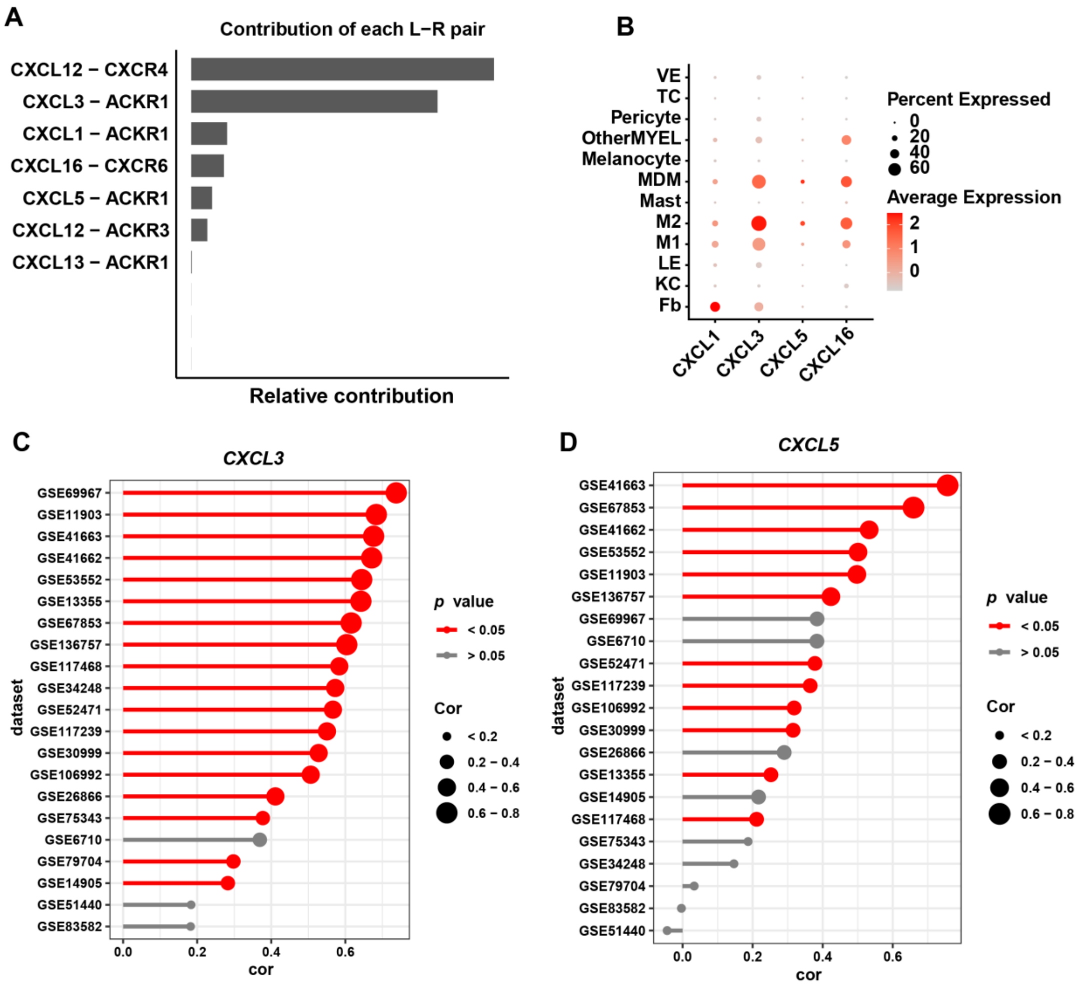


**Figure S4 High-pyroptosis MDMs mediate intercellular signaling primarily through the CXCL1/3/5/16 axis. (A)** Bar plot showing ligand-receptor pair contributions to CXCL pathway signaling in psoriasis cell communication analysis. (**B**) Dot plots depicting the comparative expression levels of CXCL1, CXCL3, CXCL5, and CXCL16 in macrophages versus other cell subtypes. (**C**) Correlation between CXCL3 expression levels and psoriasis severity across bulk-RNA datasets. (**D**) Correlation between CXCL5 expression levels and psoriasis severity across bulk-RNA datasets. VE, Vascular endothelial cells; TC, T cells; MYEL, Myeloid cells; MDM, Monocyte-derived macrophage; M, maceophages; LE, lymphatic endothelial cells; KC, Keratinocytes; Fb, Fibroblast.

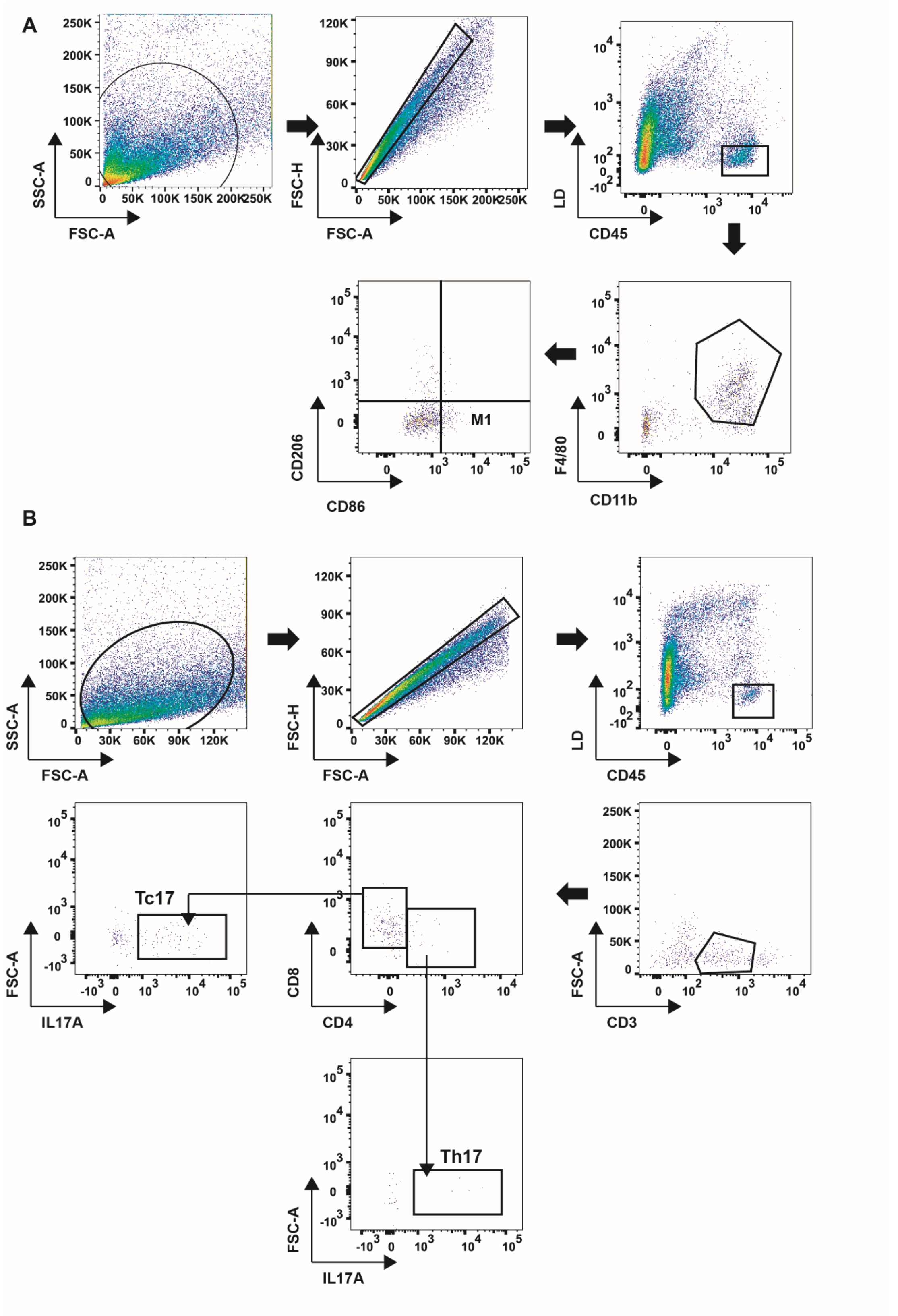


**Figure S5** Schematic of Gating for Macrophages (**A**) and Gating for Th17 and Tc17 Cells in the Skin (**B**).

**Table S1 Information about the relevant GEO data set.**

| Number | Total Sample Size | Year |
|---|---|---|
| GSE117468 | 565 | 2020 |
| GSE136757 | 105 | 2020 |
| GSE117239 | 324 | 2019 |
| GSE106992 | 192 | 2019 |
| GSE83582 | 102 | 2017 |
| GSE79704 | 64 | 2017 |
| GSE69967 | 95 | 2016 |
| GSE67853 | 27 | 2016 |
| GSE75343 | 45 | 2015 |
| GSE51440 | 59 | 2014 |
| GSE53552 | 99 | 2014 |
| GSE52471 | 38 | 2013 |
| GSE41663 | 81 | 2013 |
| GSE34248 | 28 | 2013 |
| GSE41662 | 48 | 2013 |
| GSE30999 | 170 | 2012 |
| GSE26866 | 37 | 2012 |
| GSE11903 | 89 | 2009 |
| GSE14905 | 82 | 2009 |
| GSE13355 | 180 | 2009 |
| GSE6710 | 26 | 2007 |

**Table S2 List of 57 genes associated with pyroptosis.**

| Gene | Resource | Function |
|---|---|---|
| *AIM2* | PMID:36618365 | Inflammasome |
| *APIP* | PMID: 37179249 | - |
| *BAK1* | MSigDB | - |
| *BAX* | MSigDB | - |
| *CASP1* | MSigDB | Cleave proteins |
| *CASP3* | MSigDB | Cleave proteins |
| *CASP4* | MSigDB | Cleave proteins |
| *CASP5* | MSigDB | Cleave proteins |
| *CASP6* | PMID: 35990678 | Cleave proteins |
| *CASP8* | PMID: 35990678 | Cleave proteins |
| *CASP9* | PMID: 35990678 | Cleave proteins |
| *CHMP2A* | MSigDB | Negative regulator |
| *CHMP2B* | MSigDB | Negative regulator |
| *CHMP3* | MSigDB | Negative regulator |
| *CHMP4A* | MSigDB | Negative regulator |
| *CHMP4B* | MSigDB | Negative regulator |
| *CHMP4C* | MSigDB | Negative regulator |
| *CHMP6* | MSigDB | Negative regulator |
| *CHMP7* | MSigDB | Negative regulator |
| *CYCS* | MSigDB | - |
| *DHX9* | PMID: 37179249 | Inflammasome |
| *ELANE* | MSigDB | Cleave proteins |
| *GPX4* | PMID: 35990678 | Negative regulator |
| *GSDMA* | PMID: 35990678 | Executive protein |
| *GSDMB* | PMID:36618365 | Executive protein |
| *GSDMC* | PMID: 35990678 | Executive protein |
| *GSDMD* | MSigDB | Executive protein |
| *GSDME* | MSigDB | Executive protein |
| *GZMA* | PMID: 37008939 | Cleave proteins |
| *GZMB* | MSigDB | Cleave proteins |
| *HMGB1* | MSigDB | Negative regulator |
| *IL18* | MSigDB | Negative regulator |
| *IL1A* | MSigDB | Negative regulator |
| *IL1B* | MSigDB | Negative regulator |
| *IL6* | PMID: 35990678 | Negative regulator |

| Gene | Resource | Function |
|---|---|---|
| *IRF1* | MSigDB | - |
| *IRF2* | MSigDB | - |
| *NAIP* | PMID: 23702978 | Inflammasome |
| *NLRC4* | PMID:36618365 | Inflammasome |
| *NLRP1* | PMID:36618365 | Inflammasome |
| *NLRP2* | PMID: 35990678 | Inflammasome |
| *NLRP3* | PMID:36618365 | Inflammasome |
| *NLRP6* | PMID: 23702978 | Inflammasome |
| *NLRP7* | PMID: 23702978 | Inflammasome |
| *NLRP9* | PMID: 37179249 | Inflammasome |
| *NOD1* | PMID: 35990678 | Activate the inflammasome |
| *NOD2* | PMID: 23702978 | Activate the inflammasome |
| *PJVK* | PMID: 35990678 | Executive protein |
| *PLCG1* | PMID:36618365 | Poreforming protein |
| *PRKACA* | PMID: 35990678 | - |
| *PYCARD* | PMID: 35990678 | Adapter protein |
| *SCAF11* | PMID:36618365 | - |
| *TIRAP* | PMID: 35990678 | Adapter protein |
| *TNF* | PMID: 35990678 | Activate the inflammasome |
| *TP53* | MSigDB | - |
| *TP63* | MSigDB | - |
| *ZBP1* | PMID: 37686375 | Inflammasome |

“-” represents a function that has not yet been identified in pyroptosis.

**Table S3 Marker genes defining macrophage subpopulations in human skin scRNA-seq.**

| M1 | M2 | MDM |
|---|---|---|
| *RNASE1* | *HSPA1A* | *FCER1G* |
| *FTL* | *HSPH1* | *CXCL8* |
| *CTSB* | *CXCL8* | *CXCL3* |
| *FCER1G* | *HLA-DRA* | *TYROBP* |
| *C1QC* | *HSPA1B* | *CXCL2* |
| *CTSL* | *FCGR2A* | *MMP9* |
| *TYROBP* | *EMP3* | *LYZ* |
| *C1QB* | *HSPB1* | *EREG* |
| *CD68* | *HSPA6* | *CTSB* |
| *CD163* | *TYROBP* | *SRGN* |
| *FTH1* | *DNAJB1* | *C15orf48* |
| *MS4A7* | *CD83* | *FTL* |
| *TGFBI* | *HSP90AA1* | *FTH1* |
| *SRGN* | *GPR183* | *IL1B* |
| *PLD3* | *CCL3* | *MS4A7* |
| *S100A9* | *CCL4* | *CD14* |
| *C1QA* | *HLA-DRB1* | *PLAUR* |
| *TYMP* | *CTSZ* | *PILRA* |
| *MMP9* | *CXCL2* | *TIMP1* |
| *C15orf48* | *PLEK* | *PID1* |
| *LILRB4* | *RNASE1* | *CD68* |
| *GLUL* | *GADD45B* | *PLA2G7* |
| *EMP3* | *MS4A6A* | *CXCL5* |
| *ABCA1* | *AIF1* | *THBS1* |
| *SPI1* | *FCER1G* | *ANPEP* |
| *MAFB* | *FTL* | *TNFRSF1B* |
| *CTSZ* | *FGL2* | *CTSL* |
| *HLA-DRA* | *CD68* | *RNASE1* |
| *CXCL8* | *CXCL3* | *HLA-DRA* |
| *PSAP* | *C5AR1* | *CXCL16* |

M, maceophages; MDM, Monocyte-derived macrophage.

**Table S4 The characteristics of psoriasis patients and healthy controls for detection of pyroptosis-related markers in MDMs by Western blotting.**

| No. | Gender | Age | PASI Score |
|---|---|---|---|
| Control 1 | Female | 28 | - |
| Control 2 | Male | 26 | - |
| Control 3 | Female | 28 | - |
| Control 4 | Male | 28 | - |
| Control 5 | Female | 41 | - |
| Control 6 | Female | 55 | - |
| Control 7 | Female | 30 | - |
| Control 8 | Male | 24 | - |
| Control 9 | Male | 30 | - |
| Control 10 | Male | 27 | - |
| Control 11 | Female | 25 | - |
| Control 12 | Female | 23 | - |
| Control 13 | Female | 31 | - |
| Psoriasis 1 | Female | 28 | 2.1 |
| Psoriasis 2 | Male | 26 | 8.2 |
| Psoriasis 3 | Female | 29 | 1.2 |
| Psoriasis 4 | Male | 29 | 4.9 |
| Psoriasis 5 | Female | 42 | 6.4 |
| Psoriasis 6 | Female | 55 | 7.8 |
| Psoriasis 7 | Female | 30 | 5.2 |
| Psoriasis 8 | Male | 24 | 0.4 |
| Psoriasis 9 | Male | 31 | 6.4 |
| Psoriasis 10 | Male | 28 | 8.7 |
| Psoriasis 11 | Female | 26 | 3.3 |
| Psoriasis 12 | Female | 24 | 3.1 |
| Psoriasis 13 | Female | 31 | 9.6 |
| Psoriasis 14 | Female | 29 | 14.7 |
| Psoriasis 15 | Female | 29 | 0.4 |

**Table S5 The characteristics of psoriasis patients and healthy controls for detection of pyroptosis-related markers in MDMs by RT-qPCR.**

| NO. | Gender | Age | PASI score |
|---|---|---|---|
| Control 1 | Female | 28 | - |
| Control 2 | Male | 26 | - |
| Control 3 | Female | 23 | - |
| Control 4 | Female | 41 | - |
| Control 5 | Female | 30 | - |
| Control 6 | Male | 24 | - |
| Psoriasis 1 | Female | 28 | 17.1 |
| Psoriasis 2 | Male | 25 | 6.9 |
| Psoriasis 3 | Male | 25 | 3.9 |
| Psoriasis 4 | Female | 42 | 13.7 |
| Psoriasis 5 | Female | 40 | 14.2 |
| Psoriasis 6 | Female | 31 | 6.4 |
| Psoriasis 7 | Female | 32 | 6.5 |
| Psoriasis 8 | Female | 22 | 6.2 |
| Psoriasis 9 | Female | 21 | 6.5 |
| Psoriasis 10 | Male | 25 | 6.9 |

**Table S6 The characteristics of psoriasis patients and healthy controls for detection of CXCL16 in MDMs by RT-qPCR.**

| NO. | Gender | Age | PASI score |
|---|---|---|---|
| Control 1 | Male | 46 | - |
| Control 2 | Male | 28 | - |
| Control 3 | Female | 33 | - |
| Psoriasis 1 | Female | 33 | 14.1 |
| Psoriasis 2 | Male | 25 | 7.9 |
| Psoriasis 3 | Male | 45 | 7.8 |

**Table S7 Primers for RT-qPCR.**

| Gene | Forward primer (5'-3') | Reverse primer (5'-3') |
|---|---|---|
| *Il1β-m* | GCAACTGTTCCTGAACTCAACT | ATCTTTTGGGGTCCGTCAACT |
| *Tnfα-m* | CATCTTCTCAAAATTCGAGTGACAA | CCAGCTGCTCCTCCACTTG |
| *Il6-m* | CACAGAGGATACCACTCCCAACA | TCCACGATTTCCCAGAGAACA |
| *Il22-m* | GCTGGAAGTTGGACACCTCAA | ATGAGTTTTTCCCTTATGGGGAC |
| *Il17a-m* | ACTACCTCAACCGTTCCA | GAATCTGCCTCTGAATCCA |
| *Il17c-m* | ATGCTTGTGTCGTGGATG | GTGCCTGGAATGTCTGTC |
| *Il17f-m* | TGCTACTGTTGATGTTGGGAC | AATGCCCTGGTTTTGGTTGAA |
| *Cxcl16-m* | CCTTGTCTCTTGCGTTCTTCC | TCCAAAGTACCCTGCGGTATC |
| *Cxcl1-m* | CTGGGATTCACCTCAAGAACATC | CAGGGTCAAGGCAAGCCTC |
| *Gapdh-m* | AGGTCGGTGTGAACGGATTTG | GGGGTCGTTGATGGCAACA |
| *TNFα-h* | GAGGCCAAGCCCTGGTATG | CGGGCCGATTGATCTCAGC |
| *IL6-h* | AGACAGCCACTCACCTCTTCAG | TTCTGCCAGTGCCTCTTTGCTG |
| *CXCL1-h* | AAGAACATCCAAAGTGTGAACG | CACTGTTCAGCATCTTTTCGAT |
| *CXCL16-h* | CCCGCCATCGGTTCAGTTC | CCCGCCATCGGTTCAGTTC |
| *GAPDH-h* | GGAAGATGGTGATGGGATTTC | GAAGGTGAAGGTCGGAGTCAA |

m, mouse; h, human
